# Early Warning Models to Predict the 90-Day Urinary Tract Infection Risk After Radical Cystectomy and Urinary Diversion for Patients With Bladder Cancer

**DOI:** 10.3389/fsurg.2021.782029

**Published:** 2022-01-21

**Authors:** Xun Lu, Hua Jiang, Dong Wang, Yiduo Wang, Qi Chen, Shuqiu Chen, Ming Chen

**Affiliations:** ^1^Department of Urology, Affiliated Zhongda Hospital of Southeast University, Nanjing, China; ^2^Surgical Research Center, Institute of Urology, School of Medicine, Southeast University, Nanjing, China; ^3^Department of Interventional Radiology, Affiliated Zhongda Hospital of Southeast University, Nanjing, China

**Keywords:** nomogram, urinary diversion, urinary tract infection, risk factor, prognostic nutritional index

## Abstract

**Purpose:**

To develop and validate a nomogram of the 90-day urinary tract infection (UTI) risk for patients with bladder cancer undergoing radical cystectomy (RC) and urinary diversion.

**Patients and Methods:**

The predictive nomogram was based on a retrospective study on the consecutive patients who underwent RC and urinary diversion for bladder cancer between January 2014 and March 2021. The incidence and microbiology of UTI were reported. The univariate and multivariate logistic analyses were conducted to determine independent risk factors associated with UTI. The predictive accuracy and discriminatory ability of the established nomogram were evaluated by the concordance index (C-index) and decision curve analysis (DCA). The performance of the model was validated internally.

**Results:**

A total of 220 patients were included and the incidence of UTI within 90 days was 27.3%. The most commonly identified pathogens were *Enterococcus* (42.0%), *Escherichia coli* (21.70%), and *Candida* (13.0%). Urinary diversion type, Charlson comorbidities index (CCI), stricture, and prognostic nutritional index (PNI) were included in the nomogram. The C-index of the nomogram for predicting UTI was 0.858 (95% CI: 0.593–0.953). In the validation cohort, the nomogram also showed high-predictive accuracy. Net reclassification improvement (NRI) and integrated discrimination improvement (IDI) index indicated that PNI led to improvement in predictive ability.

**Conclusion:**

The proposed early warning model shows great accuracy in predicting the incidence of 90-day UTI after RC and urinary diversion in patients with bladder cancer.

## Introduction

Bladder cancer is a primary cancer of the urothelium, with relatively high incidence and mortality worldwide (1). Muscle-invasive bladder cancer (MIBC) accounts for about 25% of the bladder cancer cases (2). Because of the high risk of recurrence and metastasis, radical cystectomy (RC) and urinary diversion is often recommended when diagnosed at the advanced stage.

In the recent decades, despite the improvement of surgical techniques and advances in preoperative management, RC and urinary diversion have still been acknowledged to be the most complicated surgery in urology (3). The associated risk of RC and urinary diversion is based not only on the technical challenges of the surgery, but also on the characteristics of the patients. Patients who underwent RC and urinary diversion are often of advanced age and accompanied by comorbidity and malnutrition. Many studies have reported common complications after RC such as incisional infection, mechanical bowel obstruction, bleeding, lymphatic leak, and ureteroenteric anastomotic stricture (4–6). The incidence of early complications after RC (within 90 days after surgery) is reported to be 20–57% (7, 8) and postoperative infection is one of the most common complications, especially urinary tract infection (UTI) (9). Considering a substitution of intestine for urinary tract reconstruction, patients who underwent urinary diversion are particularly susceptible to UTI.

To predict the common complication of UTI for an early management, researchers (6, 10–15) have made efforts to find possible risk factors and microbiology of UTI within 90 days after RC and urinary diversion. The postoperative 90-day UTI rates vary across different patient selection and urinary diversion types. Early UTI increases the readmission rate and hospitalization cost. Therefore, it is critical to predicting the probability of early UTI.

In this study, we conducted a retrospective study to analyze the risk factors of UTI within 90 days in the patients with bladder cancer after RC and urinary diversion. Moreover, the microbiology of UTI was also identified. To our knowledge, this study is the first attempt to develop and validate a predictive nomogram for this particular population to determine the possibility of incidence of UTI within 90 days after RC and urinary diversion.

## Patients and Methods

### Study Design

A retrospective study was conducted on the hospital of the authors for patients with bladder cancer who underwent RC with urinary diversion between January 2014 and March 2021. The inclusion criteria included age ≥18 years; no history of immune system diseases or other malignant tumors; and histopathologically proven urothelial carcinoma. The exclusion criteria were an active preoperative infection with a positive urine culture even after a period of use of antibiotics or a positive urinary nitrite with typical symptoms such as flank pain or fever; upper tract urothelial carcinoma; palliative or salvage cystectomy. Patients lost in the follow-up or with missing data were also excluded in the study. Baseline clinicopathologic characteristics included age, gender, body mass index (BMI), Charlson comorbidities index (CCI), smoking status, diabetes, hypertension, prognostic nutritional index (PNI), type of surgery, perioperative blood transfusion, pathological stage, previous abdominal surgery, urinary diversion type, and complications such as ureteral stent obstruction or ureteroenteric anastomotic strictures. PNI is an index obtained through peripheral blood, which reflects nutrition and inflammation status of patient. The entire cohort was then randomly divided into the training cohort and the validation cohort. In developing and validating this nomogram, we strictly followed the TRIPOD checklist (Supplementary Table 1).

This study was approved by the local institutional review board and was censored on June 30, 2021. Informed consent was waived due to the retrospective nature.

### Treatment

All the patients diagnosed with bladder cancer underwent standard RC and pelvic lymphadenectomy by two experienced surgeons at our tertiary institution. RC was conducted with open, laparoscopic or robotic assistance. For urinary diversion, continent cutaneous diversion (CCD) or ileal conduit (IC) were conducted on patients with impaired renal function, and tumors involving the prostatic urethra in male or the bladder neck in female. Orthotopic neobladder (ONB) was constructed according to the preference of the patients and their treating urologists.

All the patients included in the study received second-generation cephalosporin antibiotic prophylaxis preoperatively. Postoperatively, patients with ONB reconstruction were rinsed by sodium bicarbonate twice daily to keep drainage. Generally, ureteral stents were implanted in CCD, patients with IC and ONB in our center after surgery. For the patients who adopt CCD urinary diversion, the ureteral stent was regularly replaced every 3 months, while patients who underwent IC and ONB diversion usually removed the ureteral stents 3 months after the surgery. Each patient was encouraged for early oral feeding and mobilization. For ureteroenteric anastomosis, the Bricker techniques were performed as ureters were sutured separately onto the ileum in an end-to-side way. Urine cultures were collected after cleaning the catheter or vulva of the patients. For the patients with clinically suspected UTI, urine specimens were usually collected before the use of antibiotics. Urine specimens of other patients were collected in the morning during hospitalization. All the procedures were conducted by experienced nurses to avoid specimen contamination.

### Definitions and Outcomes

The incidence of UTI within 90 days was recorded. UTI was defined as positive urine culture (≥10^5^ cfu/ml) with or without associated fever or flank pain. The calculation of PNI was albumin level (g/L) + 5 × lymphocyte count (10^9^/L). The diagnosis of stricture was confirmed by CT or even endoscopic management when there was obvious hydronephrosis. The obstruction of J-stent was also defined as stricture in the study.

The primary outcomes in the study were the incidence and independent risk factors of UTI within 90 days. The second outcome was microbiology of UTI.

### Statistical Analysis

Continuous variables were represented by mean ± SD or median ± interquartile range (IQR) according to the distribution and compared by the *t*-test or the Mann–Whitney *U* test. Categorical variables were described using frequencies or percentages and compared using the chi-squared and Fisher's exact tests. The univariate analysis of variables was conducted to estimate potential factors associated with the incidence of UTI within 90 days. All the variables with *p* < 0.10 in univariate analysis were subsequently entered into the multivariate logistic analysis. Coefficients of multivariate logistic regression models were then used to develop the nomogram. *p* < 0.05 was considered as statistically significant.

A predictive nomogram was developed by integrating all the significant variables according to the multivariate analysis, and it was drawn by R software version 4.0.3 (http://www.r-project.org/). The performance of the developed nomogram was determined by C-index and decision curve analysis (DCA). The agreement between the predicted probability and the actual probability was measured by a calibration plot generated from the nomogram. Validation of the nomogram was performed in the validation cohort. All the statistical analysis was performed by the software Stata 15.1 (StataCorp, College Station, Texas, USA).

## Results

### Baseline Characteristics of Patients

**Figure 1:**
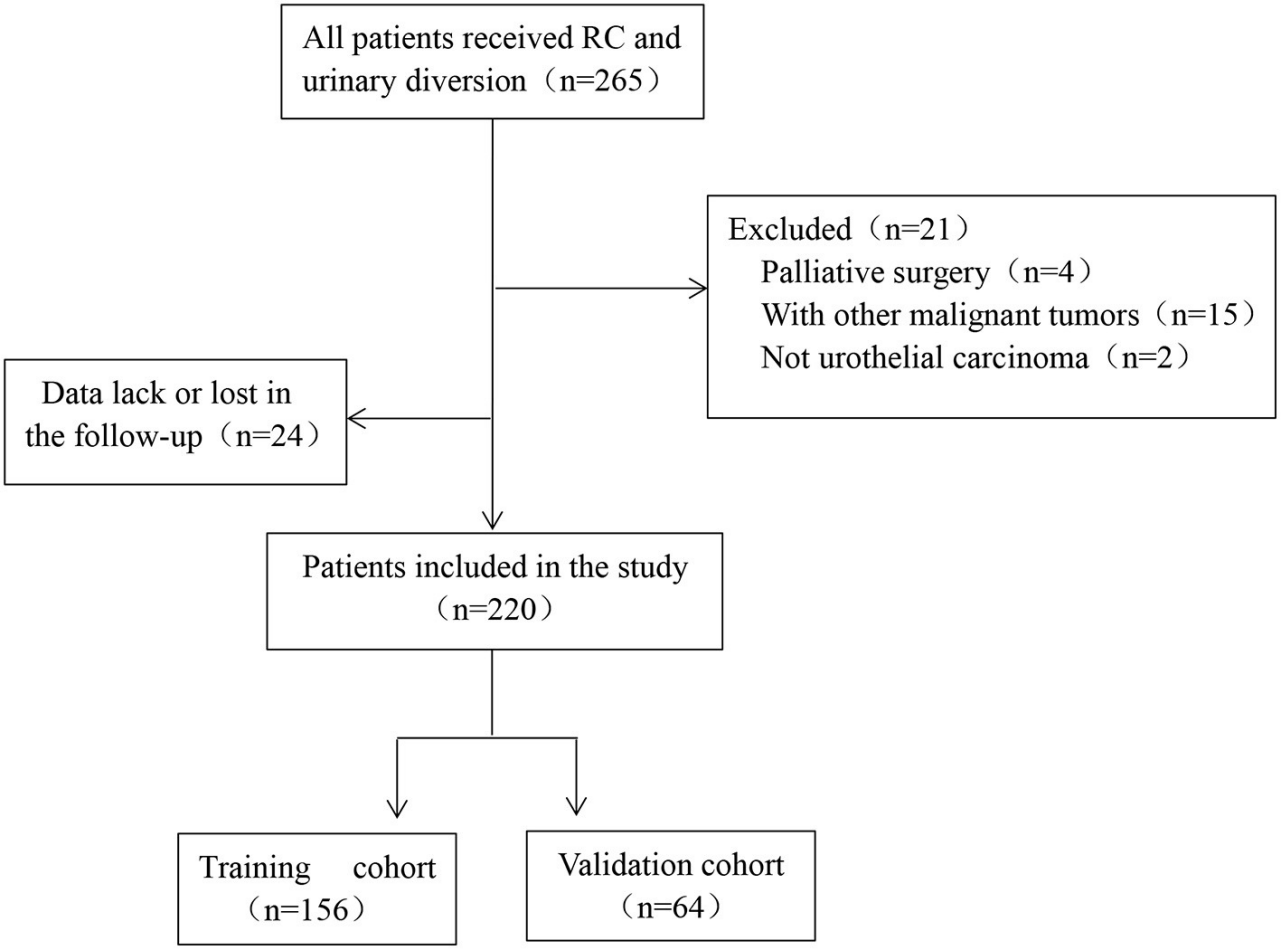
The flowchart of protocol in the study.

According to the inclusion and exclusion criteria, 220 patients with bladder cancer undergoing RC and urinary diversion were finally enrolled in the study and then divided into the training cohort and validation cohort randomly. The flow chart of protocol was shown in Figure 1.

In the training cohort, 141 (90.4%) males and 15 (9.6%) females with median age of 70 years (IQR, 61.5–75 years) were included. The preoperative levels of PNI were 45.9 (IQR, 42.0–51.0) and BMI was 24.6 kg/m^2^ (IQR, 22.3–27.0 kg/m^2^). The number of patients with stage T1-2 or stage T3-4 is 137 and 19, respectively. For the urinary diversion, 116 (74.4%) patients received CCD, 15 (9.6%) patients received IC, and 25 (16%) patients underwent ONB, respectively. The detailed clinicopathological characteristics of patients in the training and validation cohort were listed in Supplementary Table 1.

### Incidence and Risk Factors of Urinary Tract Infection

The incidence of UTI within 90 days was 27.3% among 220 patients. A total of 69 episodes were recorded in 60 patients. Among these patients, 7 patients (11.7%) had documented urosepsis and 14 patients (23.3%) had more than one episode of UTI. In the training cohort, univariate analysis indicated that gender [*p* = 0.090, odds ratio (OR) = 0.392; 95% CI: 0.13–1.16], CCI (*p* = 2.099, OR = 0.041; 95% CI: 1.03–4.28), PNI (*p* < 0.0001, OR = 0.860; 95% CI: 0.80–0.92), stricture (*p* < 0.0001, OR = 19.643; 95% CI: 5.32–72.59), diabetes (*p* = 0.092, OR = 0.380; 95% CI: 0.12–1.17) and urinary diversion type (IC: *p* = 0.049, OR = 3.029; 95% CI: 1.01–9.14; ONB: *p* = 0.072, OR = 2.308; 95% CI: 0.93–5.74) were associated with UTI among bladder cancer patients. Then, the identified variables were further entered into multivariate logistic analysis. The results showed that CCI (*p* = 0.042, OR = 2.765; 95% CI: 1.04–7.38), PNI (*p* < 0.001, OR = 0.844; 95% CI: 0.77–0.92), stricture (*p* < 0.001, OR = 17.909; 95% CI: 3.95–81.18), and urinary diversion type (IC: *p* = 0.007, OR = 6.955; 95% CI: 1.69–28.68; ONB: *p* = 0.014, OR = 4.355; 95% CI: 1.35–14.02) were independent risk factors of UTI following RC and urinary diversion (Supplementary Table 2).

### Microbiology of UTI

**Figure 2:**
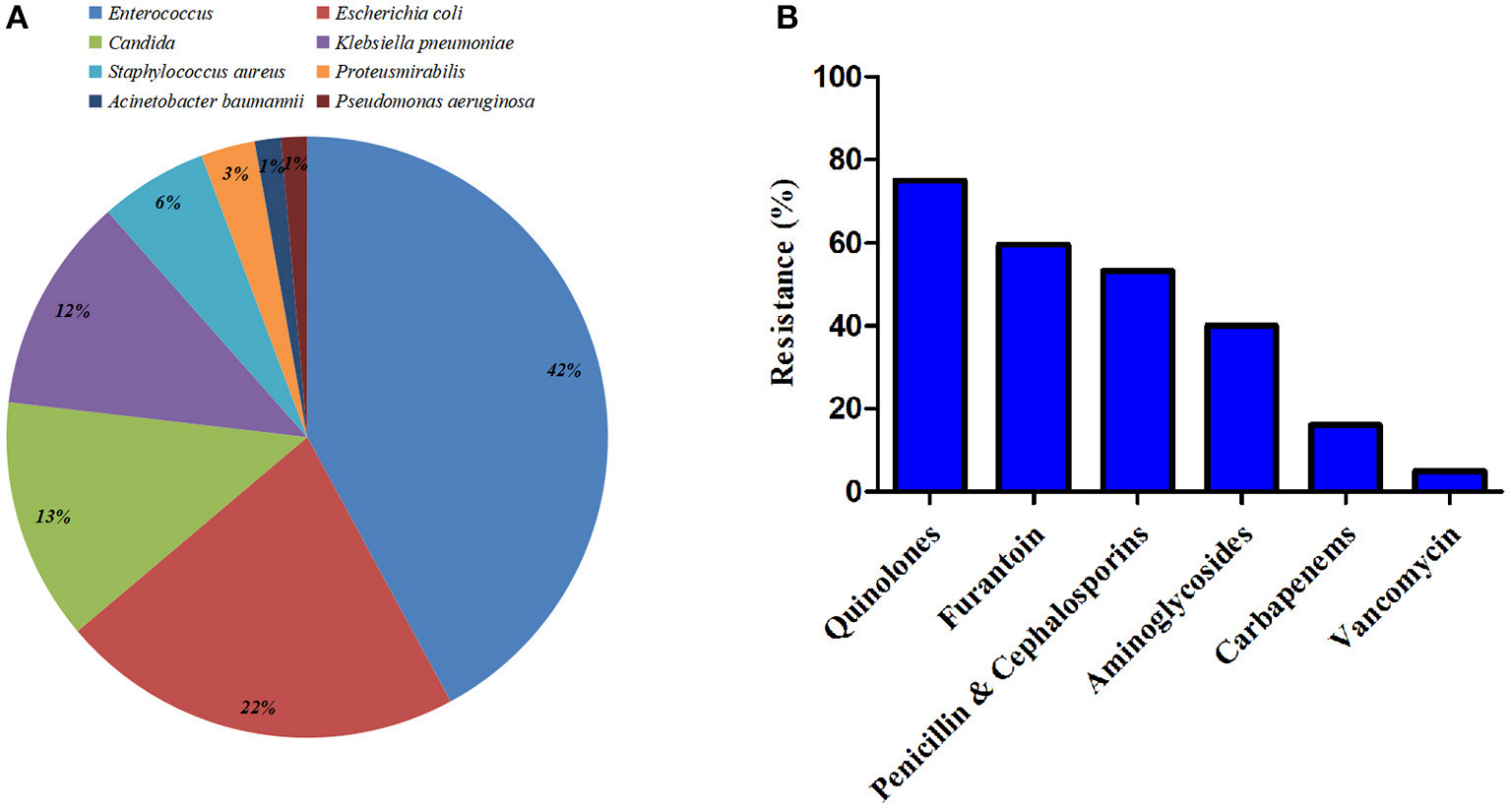
Microbiology of urinary tract infection (UTI) within 90 days after radical cystectomy (RC) and urinary diversion. **(A)** Identified pathogens among patients in the study. **(B)** Antibiotic resistance of the identified bacteria.

The microbiology of UTI was shown in Figure 2A. The most commonly identified pathogens among patients in the study were *Enterococcus* (42.0%), *Escherichia coli* (21.70%), and *Candida* (13.0%). Gram-positive accounted for 47.8%, along with 39.1 and 13.1% for Gram-negative bacteria and fungi, respectively. All the isolated pathogens of *Staphylococcus aureus* (5.8%) were methicillin-resistant *Staphylococcus aureus* (MRSA). And six producing extended-spectrum beta-lactamase (ESBL) *Escherichia coli* were identified. In addition, resistance to quinolones, penicillin and cephalosporins, and carbapenems were noted at 75.0, 53.3, and 16.0%, respectively (Supplementary Table 2). Sensitivity was the best for vancomycin in Gram-positive bacteria (93.9%). No resistance was found in fungi neither flucytosine nor amphotericin. Antibiotic resistance of identified pathogens was shown in Figure 2B.

### Development of a Predictive Nomogram for UTI

**Figure 3:**
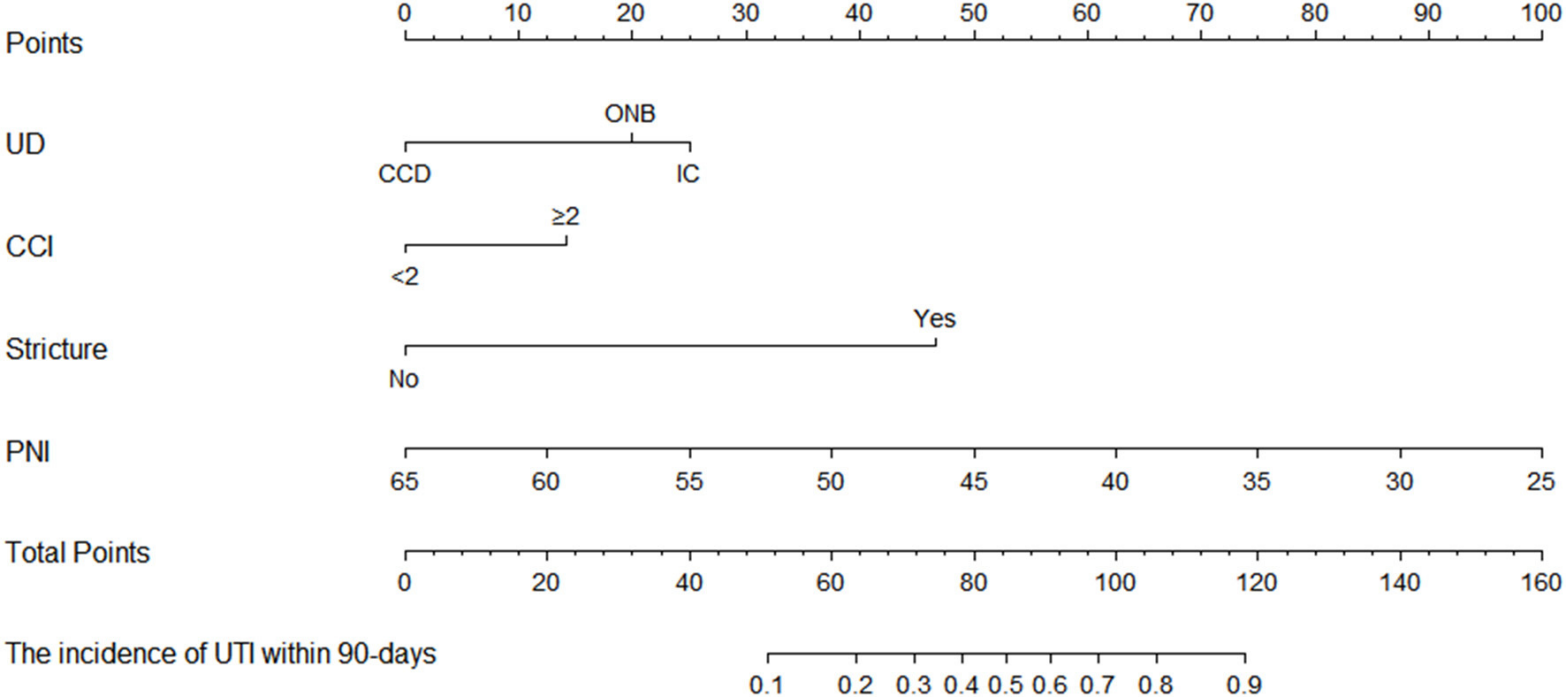
The predictive nomogram of incidence of UTI within 90 days in patients with bladder cancer underwent RC and the urinary diversion.

The established predictive nomogram for UTI in the training cohort was shown in Figure 3. Each included factor has a variety of risk points, which can be represented by drawing a vertical line directly upward from the corresponding predictive factor to an axis with a “points.” “Total points” are derived by adding respective risk points, and a vertical line can be drawn to the axis marked “The incidence of UTI within 90-days” in order to determine the possibility of incidence of UTI within 90 days of a specific patient.

**Figure 4:**
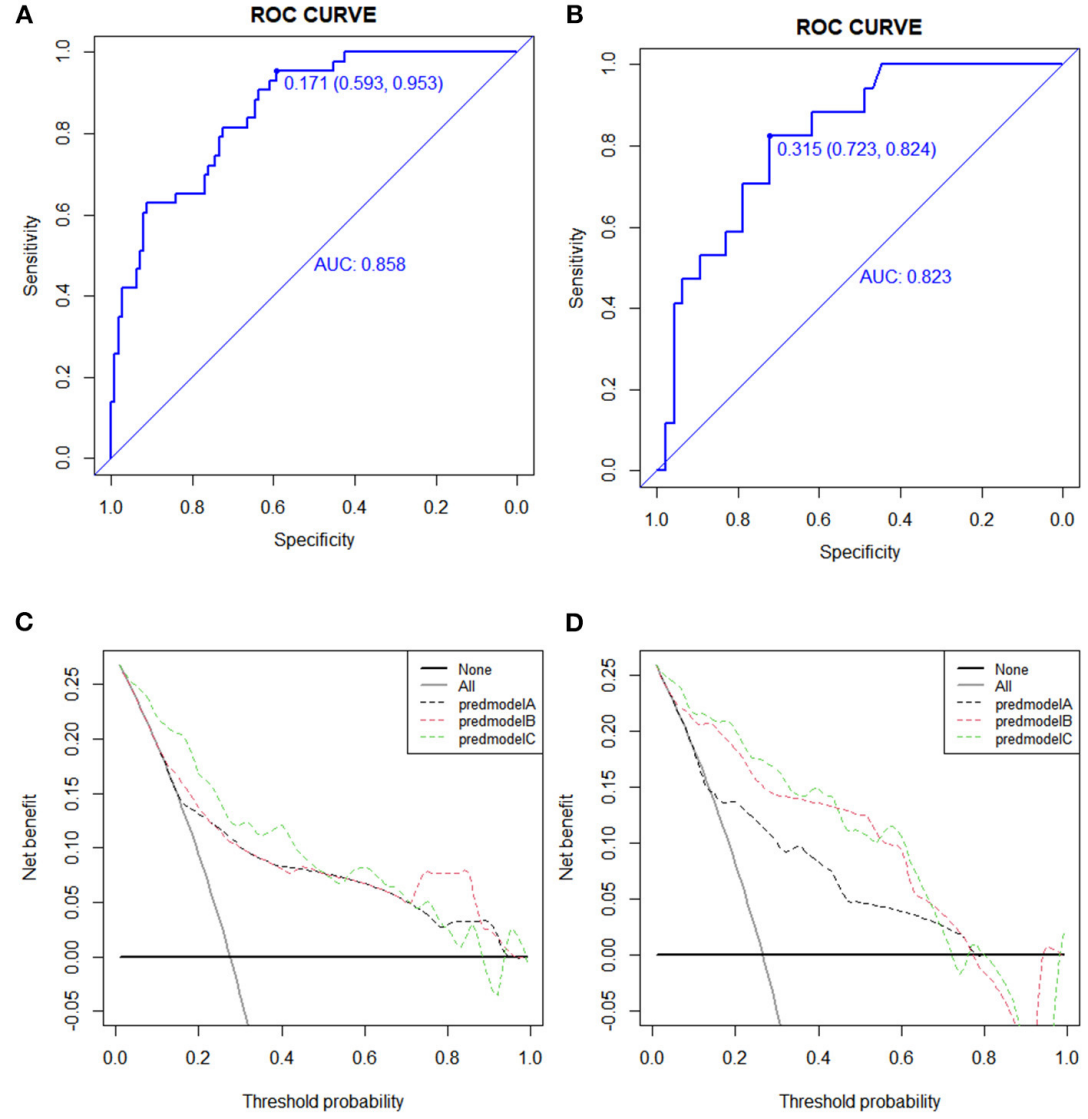
Receiver operating characteristic (ROC) and decision curve analysis (DCA) curve in the training and validation cohort. **(A)** The ROC cure in the training cohort. **(B)** The ROC curve in the validation cohort. **(C)** The DCA cure in the training cohort. **(D)** The DCA curve in the validation cohort.

**Figure 5:**
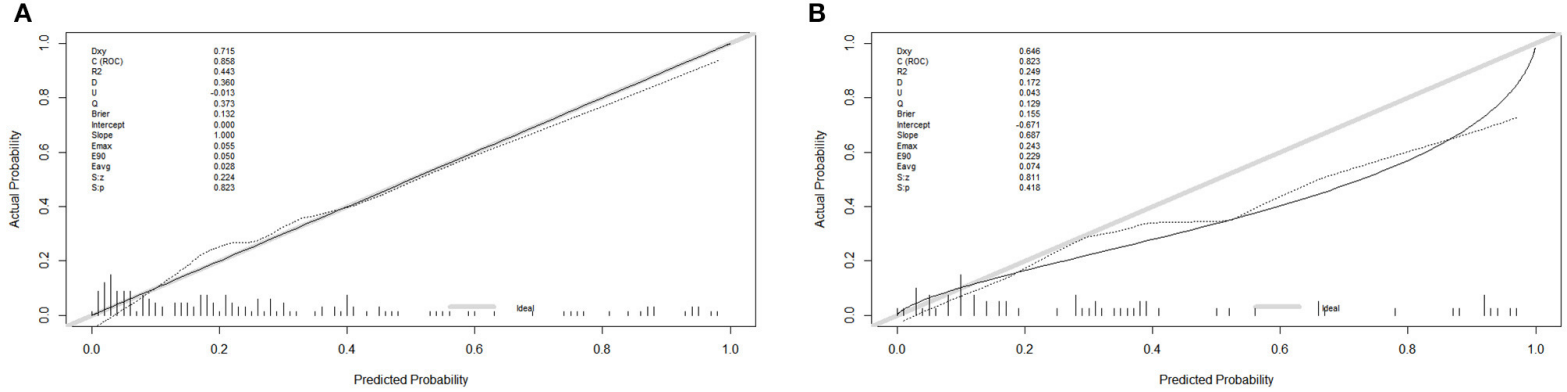
The calibration plot in the training and validation cohort. **(A)** The calibration plot in the training cohort. **(B)** The calibration plot in the training cohort.

In the training cohort, the receiver operating characteristic (ROC) curve was shown in Figure 4A and the C-index for UTI was 0.858 (95% CI: 0.593–0.953). The calibration plot showed a great agreement between predicted and actual probability (Figure 5A).

### Validation of a Predictive Nomogram for UTI

In the validation cohort, the ROC curve was shown in Figure 4B. The C-index was 0.823 (95% CI: 0.723–0.824), which represented high-predictive accuracy. The calibration curve also showed good accordance between the predicted and actual probability of incidence of UTI within 90 days (Figure 5B).

### Decision Curve Analysis

The decision curve for the predictive nomogram for UTI in the training and validation cohort was shown in Figures 4C,D. In the study, net reclassification improvement (NRI) and integrated discrimination improvement (IDI) were used to evaluate the potential benefit of the developed nomogram. In Table 1, the NRI and IDI suggested that PNI improved net benefit.

**Table 1:** A comparison of discriminatory ability of model B and model C with model A using NRI and IDI in the training and validation cohort.

	**Training cohort**				**Validation cohort**			
	**NRI**	**P**	**IDI**	**P**	**NRI**	**P**	**IDI**	**P**
B vs. A	−7.4%	0.325	2.0%	0.097	11.6%	0.243	17.7%	<0.001
C vs. A	14.9%	0.102	10.7%	<0.001	22.4%	0.046	21.7%	<0.001
C vs. B	22.3%	0.006	8.7%	<0.001	10.8%	0.344	4.0%	0.114

*Model A, UD + Stricture; Model B, UD + Stricture + CCI; Model C, UD + Stricture + CCI + PNI*.

*NRI, Net Reclassification Improvement; IDI, Integrated Discrimination Improvement*.

## Discussion

Postoperative UTI is very common in patients who underwent RC and urinary. Several inherent risk factors could account for the postoperative infectious complications. One reason is that urinary diversion operation often involves the intestine. The urinary tract reconstruction often attaches part of the intestine to the upper urinary tract, especially in patients undergoing IC and ONB. The second reason is the colonization of bacteria caused by the ureteral stent indwelling. Accurate predictions about the UTI events after RC and urinary diversion are important for timely intervention. Thus, a predictive nomogram was developed, and included risk factors that were easy to acquire before surgery. Importantly, the performance of our model was also supported by the C-index and the calibration curve with the great discriminatory ability and high-predictive accuracy.

The UTI rate was relatively high in this study compared with the previous reports (27.3 vs. 9–10.3%) (6, 10, 11, 13, 15). One potential reason for this is the different definitions we used for UTI. In the aforementioned reports, UTI is defined as positive urine culture with clinical symptoms, while in our study, patients with asymptomatic bacteriuria were also included. Generally, a positive urine culture alone would not be regarded as clinically significant without accompanying fever or clinical complaints. However, patients who underwent urinary diversion with a positive urine culture are considered to be complicated UTI according to the EAU guideline. For complicated UTI, clinical treatment is difficult. It is more likely to progress to systemic and severe infection. As a result, all urologists should pay more attention to such infections and use antibiotics rationally.

With respect to the distribution of microbiology, most studies (11, 13, 14, 16) reported a predominance of Gram-negative bacteria. However, our result clearly showed a high rate of Gram-positive species in isolated cultures. In addition, we noted that fungi account for a relatively high rate (13.9%). Parker et al. (10) similarly report high rates of *Enterococcal* species (34.5%), *Staphylococcus aureus* (17.7%), as well as *Escherichia coli* (17.7%). With the application of antibiotics worldwide, the bacterial spectrum may vary over time. Notably, the frequency of antibiotic prophylaxis usage may lead to particular resistance of bacteria. In this study, the detailed analysis of the microbiology and antibiotic resistance highlights the importance of broadening antibiotic prophylaxis for the patients undergoing RC and urinary diversion. Meanwhile, it cannot be ignored of antimycotic medication in this particular population.

In this study, the univariate and subsequent multivariate logistic analyses determined that CCI, PNI, stricture, and urinary diversion type are predictors of UTI. Therefore, a nomogram is presented concisely with these factors. Other studies have reported different risk factors including diabetes (10), BMI (17, 18), female gender (19), urine leakage, and receipt of a perioperative blood transfusion (10). Kim et al. (16) indicated that only ureteral stricture was independent risk factor of UTI (*P* = 0.023, OR = 5.93; 95% CI: 1.28–27.52). In fact, the stricture is a well-known risk factor for UTI. The reported rate of strictures after ureteroenteric anastomotic is about 10% (20), and most of which is probably caused by periureteral fibrosis or scarring secondary to ischemia after surgery (5, 21, 22). The formation of stricture contributed to infection, hydronephrosis, and even kidney failure (23). Therefore, for these patients, more closely follow-up and timely intervention are needed in order to preserve the renal function.

Controversy remains between the urinary diversion type and UTI risk. Parker et al. (10) proposed that continent urinary diversion resulted in significantly increased UTI risk. Likewise, a population-based study (15) in Sweden showed that patients who underwent ONB diversion have an increased risk of UTI compared with those who received IC reconstruction (OR = 1.21; 95% CI: 1.05–1.39). However, Clifford et al. (13) concluded that no significant difference in the 90-day UTI rate between the three diversion types (ONB, IC, and CCD). In this study, we noted that compared with CCD, IC, and ONB reconstruction increased UTI risk (OR = 6.955; 95% CI: 1.69–28.68; and OR = 4.355; 95% CI: 1.35–14.02, respectively). A potential explanation for this association may be the prolonged duration of indwelling urinary catheters in patients after ONB reconstruction. Lo et al. (24) demonstrated that indwelling a urinary catheter for every additional day increased the risk of UTI by 3–7%. Although IC seems to contribute more to UTI than ONB, it cannot be underestimated that the cumulative incidence of UTI in patients with ONB reconstruction increased over the time.

Interestingly, PNI, a nutrition and inflammation-related index was identified as a predictor for UTI in our nomogram. Previous studies (25–27) have evaluated the prognostic value of PNI in many cancers. To date, no studies have explored the predictive ability of PNI in UTI. In this work, we demonstrated that a comprehensive model comprised of PNI enhanced the predictive ability of UTI, as shown by improvement in NRI and IDI. Traditionally, BMI and preoperative serum levels of albumin were used to predict the risk of UTI. The calculation of PNI is based on preoperative albumin levels and our findings support prior research that preoperative nutrition status could be a predictor of postoperative UTI.

Urinary tract infection had a great impact on the length of hospital stay and increased morbidity and cost after RC (28). Several efforts have been made to prevent this common postoperative complication. Large et al. (29) tried to abrogate mechanical bowel preparation in patients who underwent RC and urinary diversion, however, the results showed no significant difference between the two groups in UTI rate (*p* = 0.6). Kolwijck et al. (30) suggested that the antibiotic prophylaxis should be broadened for patients undergoing RC based on relatively high incidence of infection (18.4%), especially when the ureteral stent was removed. Wang et al. (31) found that based on the results of postoperative urine culture of stub of J-stent culture in 3 and 7 days after surgery, antibiotic prophylaxis usage significantly decreased the UTI rate (*p* = 0.031).

There are several limitations of this study. First, this study was confined by its retrospective nature. Second, the nomogram was established based on data obtained from a single institution in China and was validated internally, restricting the promotion of our developed model. Third, the results of antibiotics susceptibility experiments exist *in vitro*, we are unable to detect the efficacy and side effect of selected drugs. Therefore, more well-designed prospective studies and multicenter studies are warranted.

## Conclusion

In conclusion, this study introduces an early warning model to predict the incidence of 90-day UTI after RC and urinary diversion in patients with bladder cancer. The nomogram shows great predictive accuracy.

## Data Availability Statement

The raw data supporting the conclusions of this article will be made available by the authors, without undue reservation.

## Ethics Statement

This study was approved by the Local Institutional Review Board and was censored on June 30, 2021. Informed consent was waived due to the retrospective nature.

## Author Contributions

XL, HJ, and DW conceived, designed the experiments and contributed to the interpretation of the data. XL, YW, QC, and SC participated in the experiments and drafted the manuscript. QC and XL performed the statistical analysis. SC and MC revised the manuscript. All authors read and approved the final version of the manuscript.

## Funding

This study was supported by the National Natural Science Foundation of China (Nos. SQ2017YFSF090096 and 82070773) and the Natural Science Foundation of Jiangsu Province (BE20201271).

## Conflict of Interest

The authors declare that the research was conducted in the absence of any commercial or financial relationships that could be construed as a potential conflict of interest.

## Publisher's Note

All claims expressed in this article are solely those of the authors and do not necessarily represent those of their affiliated organizations, or those of the publisher, the editors and the reviewers. Any product that may be evaluated in this article, or claim that may be made by its manufacturer, is not guaranteed or endorsed by the publisher.
