# Supplementary material for: Early Warning Models to Predict the 90-Day Urinary Tract Infection Risk After Radical Cystectomy and Urinary Diversion for Patients With Bladder Cancer
Source: Front Surg. 2022 Jan 21;8:782029. doi: 10.3389/fsurg.2021.782029 (PMC8814316; doi:10.3389/fsurg.2021.782029)
Supplement: Supplementary file 2 [file Table_2.docx]

**Supplementary table 2** Antibiotic resistance of identified bacteria

|  | Isolated bacterial | | | | | | | |
| --- | --- | --- | --- | --- | --- | --- | --- | --- |
| Antibiotics | *Enterococcus* | *E.coli* | *Candida* | *K.pneumoniae* | *Staphylococcus* | *Proteusmirabilis* | *baumannii* | *Pseudomonas aeruginosa* |
|  | S R | S R | S R | S R | S R | S R | S R | S R |
| Quinolones | 3 26 | 6 9 | NA NA | 3 5 | 1 3 | 1 1 | 0 1 | 1 0 |
| Vancomycin | 27 2 | NA NA | NA NA | NA NA | 4 0 | NA NA | 1 0 | NA NA |
| Penicillin & Cephalosporins | 6 23 | 13 2 | NA NA | 5 3 | 0 4 | 2 0 | 1 0 | 1 0 |
| Carbapenems | NA NA | 13 2 | NA NA | 6 2 | NA NA | 2 0 | NA NA | 1 0 |
| Aminoglycosides | 14 15 | 11 4 | NA NA | 6 2 | 3 1 | 1 1 | 0 1 | 1 0 |
| Furantoin | 10 19 | 7 8 | NA NA | NA NA | 3 1 | 0 2 | 1 0 | 0 1 |
